# Captive Asian short-clawed otters (*Aonyx cinereus*) learn to exploit unfamiliar natural prey

**DOI:** 10.1098/rsos.211819

**Published:** 2022-06-08

**Authors:** Alexander M. Saliveros, Madison Bowden-Parry, Fraser McAusland, Neeltje J. Boogert

**Affiliations:** Centre for Ecology and Conservation, College of Life and Environmental Sciences, University of Exeter, Penryn, Cornwall TR10 9FE, UK

**Keywords:** extractive foraging, problem-solving, otters, individual learning, social learning, network-based diffusion analysis

## Abstract

Foraging plays a vital role in animal life histories, and learning whether unfamiliar food items are palatable is a key part of this process. Animals that engage in extractive foraging must also learn how to overcome the protective measures of their prey. While otters (subfamily Lutrinae) are a taxon known for their extractive foraging behaviour, how they learn about prey palatability and acquire extractive foraging techniques remains poorly understood. Here we investigated (i) how captive Asian short-clawed otters (*Aonyx cinereus*) learned to interact with, and extract meat from, unfamiliar natural prey and (ii) how their exploitation of such prey compared to their ability to overcome artificial foraging tasks containing familiar food rewards. Network-based diffusion analysis showed that otters learned to interact with unfamiliar natural prey by observing their group mates. However, once interacting with the prey, they learned to extract the meat mainly asocially. In addition, otters took longer to overcome the protective measures of unfamiliar natural prey than those of extractive food puzzles. Asian short-clawed otter populations are declining in the wild. Increasing our understanding of how they learn to overcome novel foraging challenges could help develop pre-release training procedures as part of reintroduction programmes for otter conservation.

## 1. Introduction

Foraging, the process by which animals find and consume food, plays a critical role in their life histories [1,2]. A key part of this process in many species is learning whether unfamiliar potential food items are palatable [3–10]. There are broadly two learning strategies by which animals do this; the first is ‘asocial learning’, where they learn by sampling novel food items for themselves through processes like ‘trial-and-error’ [11,12]. The second strategy, termed ‘social learning’, involves animals learning by gathering information from others, rather than directly from the environment [4,11,13,14]. Social cues about food can be acquired through different modalities. Rats (*Rattus norvegicus domestica*), for example, prefer novel food types that they have smelled on the breath of other rats [4,15]. By contrast, various bird species use socially mediated visual cues during food selection; for example, they choose to eat food sources of the same colour as conspecifics (red-winged blackbirds, *Agelaius pheoniceus*, [16,17]; house sparrows, *Passer domesticus*, [18] and Burmese red junglefowl, *Gallus gallus spadiceus*, [4,19,20]). Although asocial and social learning strategies are distinct in the information sources they use, employment of these strategies does not have to be mutually exclusive [13,21]; animals can apply both in combination, which is often more efficient than relying exclusively on either asocial or social learning, particularly when learning to overcome complex novel foraging challenges [22–24].

One type of complex foraging challenge is presented by food items where the palatable aspects are protected by unpalatable physical measures, which make them ‘not-ready-to-eat’ without some form of processing [4,25–27]. Protective measures may involve physical adaptations such as hard shells and spines, or behavioural adaptations where prey species reside in areas that are difficult for predators to access [4,25–31]. The extractive foraging challenge presented by these food types is twofold [4,26]: foragers must learn both to recognize that such ‘defended’ food items are palatable, and to overcome the protective measures to extract the food within [4,28,32–34]. As extractive foraging often requires highly developed and dexterous processing skills, much of the research into the learning mechanisms underlying extractive foraging, and the associated decision-making processes, has focused on primates [25–28,32–36]. Juvenile and young adult white-faced capuchins (*Cebus capucinus*), for example, learn to forage on fruits and seeds encased in hard woody husks by copying the food choices of older capuchins [35,36]. They then learn how to extract these fruits and seeds from the husks via a combination of trial-and-error and by imitating the extraction techniques of conspecifics [32–34,37].

Primates are not the only taxon known for their dextrous object manipulation and extractive foraging, however. Otters (subfamily Lutrinae) are another clade renowned for their dextrous object handling across various contexts, including extractive foraging and object play [38–42]. With regard to extractive foraging, much of the research has focused on sea otters (*Enhydra lutris*) that use ‘anvil’ stones to crack open the protective shells of the mussels they feed on [43–49]. Although there is some evidence to suggest that juvenile sea otters copy the tool use and prey selection behaviours exhibited by their mothers [44,49], detailed studies of how otters learn to select and extract prey are limited. Even less is known about the acquisition of extractive foraging skills in other otter species, although see [50,51] for research on the learning of hunting behaviour in Eurasian otters (*Lutra lutra*).

Asian short-clawed otters (*Aonyx cinereus*) are one such species that employ extractive techniques to break open the hard shells of crustaceans and molluscs to extract the meat that makes up most of their diet in the wild [38,52–54]. Their highly dextrous short-clawed paws (relative to other otter species) enable them to manipulate and pry open the shells of their prey, while their rear-most upper teeth are larger than their other teeth so they can crunch through the exoskeletons of prey such as crabs [38,52,54]. Due to a reduction in their wild prey brought about by overfishing, pollution and habitat loss [52,55,56], Asian short-clawed otter populations are decreasing in the wild, and they are classified as ‘Vulnerable’ on the International Union for Conservation of Nature (IUCN) Red List of Threatened Species [52]. Furthering our understanding of otter foraging behaviour may help inform conservation action in the future [57]. In particular, detailed information on how otters learn extractive foraging skills vital for their survival in the wild could be used to develop pre-release training procedures as part of species reintroduction programmes [57–59].

Due to their elusive nature and the logistical challenges of studying Asian short-clawed otters in the wild, little is known about how they learn to recognize their hard-shelled prey items as palatable, or how they learn to extract food from them. The majority of research into their extractive foraging capabilities has involved presenting captive otters with artificial food puzzles [39,57,60–64]. In captivity, Asian short-clawed otters are commonly provisioned with food items that require no extractive techniques to eat, such as day-old chicks and mince meatballs [63,65]. Here, we investigated how two groups of captive Asian short-clawed otters, which had little to no prior experience of their natural prey, learned to recognize and overcome the extractive foraging challenges presented by three different unfamiliar natural prey types. We then combined these data with data collected when we previously presented the same otters with novel artificial food puzzles that were baited with their regular food [57]. This allowed us to compare the time it took these otters to overcome mechanistically novel extractive foraging challenges, when the food within was familiar (i.e. food puzzles with meatballs) versus unfamiliar (i.e. fish, crabs and mussels).

Our past work showed that captive groups of Asian short-clawed otters learned to interact with, and extract the food rewards from, novel foraging puzzles via a combination of individual trial-and-error (i.e. asocial) learning, and learning by observing the behaviours of others (i.e. social learning; [57]). We therefore predicted that (i) they would do the same here when learning to both interact with, and extract meat from, the natural prey items. Our previous study also indicated that otters relied more on social information when learning to interact with puzzles than when extracting the food rewards. These results suggest that otters' unfamiliarity with the puzzle boxes may have led them to look for guidance from their group mates when deciding to interact with them [57]. Then, once they had started to interact with the puzzle boxes, they recognized the food rewards inside and learned to extract them mainly asocially [57]. Due to their unfamiliarity with both the palatability and the physical protective measures of the natural prey presented here, we predicted that (ii) the captive otters would rely on social learning to a similar extent when learning to interact with, and extract meat from, natural prey items. Finally, we predicted that (iii) having to determine the palatability of the unfamiliar natural prey items upon interacting with them, would mean that otters would take more time to extract the unfamiliar meat from the novel natural prey items than to extract the familiar food rewards from the artificial foraging puzzles.

## 2. Methods

### 2.1. Study population

All data were collected from two captive groups of Asian short-clawed otters at Newquay Zoo (‘Newquay’; Cornwall, UK – 50.41° N, 05.07° W) and Tamar Otter and Wildlife Centre (‘Tamar’; Cornwall, UK – 50.69° N, 04.42°W), respectively, between November 2017 and April 2018. Both otter groups were families consisting of two parents and their various litters (*n* = 12 individuals each; subsequently *n* = 8 individuals at Newquay; see electronic supplementary material, section 1 and table S1).

### 2.2. Experimental procedure

We initially observed the otter groups to collect social association data to generate a social network for each group, which would later be used to infer the strategies adopted by the otters when learning to extract meat from novel prey items via network-based diffusion analysis (NBDA; see §2.6.2). We then presented the otter groups on different days with five different artificial foraging tasks of varying complexity, and four months later, with three different natural prey types that varied in the complexity of their protective measures. The data on social networks and artificial foraging tasks presented here have been published previously in Saliveros *et al.* [57], while we collected new data for NBDA from the ‘medium abundance experimental food presentations' described in Bowden-Parry *et al*. [66]. Here, we provide an overview of the relevant methods described in detail in Saliveros *et al.* [57] and Bowden-Parry *et al*. [66]. We then proceed to combine these two datasets to compare the same otters' extractive foraging performance when presented with novel artificial foraging tasks containing familiar food rewards versus unfamiliar natural prey.

### 2.3. Association networks

Each otter group was observed for a total of 15 separate 1 h periods over 10 consecutive days. We recorded which otters were associating at 5 min intervals during each hour (following [57,60]). We considered otters to be associating if they were within one body length of each other [67]. Individuals were identified by characteristic facial features and differences in body shape and size [57,60]. We used these observations of social associations to produce a matrix of the total number of 5 min sampling intervals where each dyad in each group was associating. Then, using the simple ratio index, we calculated an association index for each dyad [57,60,68]: the total number of 5 min intervals where each otter dyad was associating was divided by the sum of (i) the total number of 5 min intervals where that dyad was *not* associating, plus (ii) the total number of 5 min intervals where that dyad *was* associating [68]. The association indices for each dyad formed the association network for the group to which they belonged (see electronic supplementary material, section 2 for network heterogeneity information). While the 5 min sampling intervals during each hour of the 15 h observation period were not independent of one another, the number of sampling points used to calculate the association indices (and subsequently the association network) is not included in the NBDA that we employed to examine social learning (see §2.6.1). Therefore, the non-independence of the sampling points should not bias the results [57,60,69].

### 2.4. Presentation of extractive foraging challenges

#### 2.4.1. Artificial foraging tasks

The first type of foraging challenge we presented to the otters was artificial foraging tasks. We constructed five types of puzzle box out of clear plastic Tupperware containers (based on apparatus in [70]) to resemble the kind of foraging challenges Asian short-clawed otters may encounter in the wild, such as extracting meat from hard-shelled organisms, which they find by sifting through silt and reaching into the crevasses between rocks [54]. Each task type presented a different novel foraging challenge from which otters had to extract a 15 g raw beef mince meatball (electronic supplementary material, table S2). The meatballs formed part of the otters' regular diet, and they were able to see them in the transparent Tupperware containers and smell them through various openings in each task type (electronic supplementary material, table S2). The task types varied in assumed difficulty and were numbered to reflect this; task ‘1’ was assumed to be the easiest to extract the food reward from and task ‘5’ was assumed to be the most difficult (electronic supplementary material, table S2; [57]). All five task types presented mechanistically new extractive foraging challenges to the otters; although these otters had previously been presented with food puzzles as part of captive enrichment programmes, they were of a different design (Tupperware containers with either screw or clip off lids; like those presented in [60]) to those presented here.

We presented the otter groups with one task type per day for five consecutive days in the week following the collection of the social network data. During each ‘task extraction trial’, otters were presented with multiple identical replicates of the same task type. We always placed three more task apparatus than the number of otters in the group into the enclosure, so that all otters had the opportunity to extract the food reward from a task and observe their group mates doing the same [60]. To control for potential order effects, task types were presented to the two otter groups in a different, random, order. We presented the tasks at approximately 10.00 on each trial day, between the otters' regular morning and midday feeds. The equivalent weight of food used in the task rewards was left out of the otters’ morning feed to prevent overfeeding. Prior to the start of each task extraction trial, animal keepers at each wildlife centre locked all the otters in the indoor area of their enclosures so that we could place the task apparatus outside in the main enclosures. The start of each trial was signified by the release of the otters from the indoor area of their enclosures to the main outdoor area. The end of each trial was marked by the extraction of the last food reward from the last task apparatus [57].

#### 2.4.2. Natural prey

Four months after the task extraction trials (as constrained by access to the otter groups), we presented the otters with prey types that resemble those encountered by Asian short-clawed otters in the wild. These ‘natural prey types’ were rainbow trout (*Oncorhynchus mykiss*), shore crabs (*Carcinus maenas*) and blue mussels (*Mytilus edulis*), henceforth referred to as ‘fish’, ‘crabs’ and ‘mussels’, respectively. They were chosen based on their availability in the UK, and on the fact that the difficulty of extracting the meat from each prey type was assumed to be different. The fish were considered the easiest to exploit as they possessed no hard shell or exoskeleton to protect the meat, the crabs had a protective exoskeleton but with several weak points where the legs and claws joined the cephalothorax, and finally the mussels had the most difficult shell to break open, as the otters were required to pry them apart along one seam [66]. Although none of these prey species is sympatric to Asian short-clawed otters in the wild, they are morphologically similar to fish, crab and mollusc species that wild Asian short-clawed otters are known to prey on [38,52–54]. The crabs and mussels were novel to both otter groups, whereas the fish represented a non-regular food source to the Tamar group and a novel food source to the Newquay group. The fish were cut into five *ca* 30 mm wide pieces, the crabs were *ca* 80 mm in diameter, while the mussels were *ca* 100 mm in length [66].

The otter groups were subject to one ‘prey extraction trial’ per day, where we placed multiple prey items of the same type into the enclosure, where the number of prey items matched the number of otters in the group. Prey types were presented in the same order to both otter groups, with a gap of 2–6 days between each trial. We presented the prey types between 13.00 and 16.00, at least 1 h after the otters' regular midday feed (see electronic supplementary material, section 3; [66]). Before the start of each trial the wildlife centres’ animal keepers locked all the otters in the indoor area of their enclosures so that we could place the prey items outside in the main enclosures. The release of the otters from the indoor area of their enclosures to the main outdoor area marked the start of each trial. The trials were considered to have ended when the meat had been extracted from all the prey items [66].

### 2.5. Video coding

We filmed all the task and prey extraction trials from a minimum of two different angles with Panasonic HC-V380 camcorders to ensure that all the otter interactions with every task apparatus and prey item were captured on film [57,66]. For each trial, we reviewed the footage and recorded the times (to the nearest second) at which otters first interacted with, and extracted the meat from, each natural prey type. We defined first interactions as the first physical contact otters had with a prey item that still contained all the food. An otter was judged to have ‘extracted’ the food from a prey item (or task apparatus; see below) that still contained all the food when it was first observed eating the meat. We then mapped these first interaction and extraction data onto the social networks (see §2.3) and used NBDA (see §2.6.1) to examine whether otters learned to interact with, and extract the meat from, the natural prey types by copying each other [71], as we previously found for the artificial foraging tasks [57]. We also recorded the amount of time (to the nearest second) that each otter spent interacting with task apparatus or prey items before first extracting food; we termed this ‘extraction time’. We defined ‘interacting’ as any physical contact otters had with a task apparatus or prey item that still contained all the food. Any interactions with tasks or prey items where some but not all of the food had been consumed were not recorded. We then compared otters' extraction times for artificial tasks versus natural prey types, using a generalized linear mixed model (GLMM; see §2.6.2).

### 2.6. Data analyses

All data analyses were performed in R v. i368 4.0.2 [72].

#### 2.6.1. Do Asian short-clawed otters copy each other when learning to exploit natural prey items?

To examine whether Asian short-clawed otters learned to interact with, and extract the meat from, the natural prey types by copying each other, we modelled the first-time-to-interact- and extract-data from both otter groups using the time of acquisition diffusion analysis (TADA) variant of network-based diffusion analyses (NBDA; R package ‘NBDA’; [71,73]). TADA infers the transmission of information via social learning when the time sequence in which individuals first display new behaviours (e.g. interacting with, or extracting the meat from, the prey types here) follows the strength of the associations between individuals in a group's association network [71,74]. We employed a model comparison procedure, whereby we compared social and asocial learning models, while simultaneously controlling for individual-level variables (ILVs) that may have affected learning rates. By fitting parameters that control for the effects of ILVs on rates of social and asocial learning, NBDA can gauge how these learning strategies might interact [71,75]. The ILVs in our models were otter age (adults, i.e. otters aged greater than or equal to 1 year, coded as ‘1’, and pups, otters aged less than 1 year, coded as ‘0’) and sex (females coded as ‘1’ and males coded as ‘0’; see electronic supplementary material, section 4), following [57].

We fitted three types of learning models that each controlled for the effects of ILVs differently. ‘Additive’ models, in which the asocial and social learning rate estimates are summed, are fitted with a parameter that assumes ILVs only affect asocial learning rates [71,75]. Thus, when additive models best fit the data, it indicates that social and asocial learning are independent stochastic processes, and therefore individuals develop new behaviours through either one learning method or the other. Conversely, ‘multiplicative’ models, where social and asocial learning rate estimates are multiplied, are fitted with parameters that assume that both social and asocial learning rates are affected by ILVs [71,75]. Thus, when these models best fit the data, it is inferred that individuals develop novel behaviours via a combination of both social and asocial learning. Lastly, when models not fitted with any ILVs, i.e. ‘no-ILV’ models, best fit the data, it suggests that neither social nor asocial learning rates are affected by ILVs. We fit the various combinations of ILVs (i.e. age only, sex only, age and sex, or none in the case of no-ILV models) to each kind of model (i.e. asocial, social additive, multiplicative and no-ILV). To test whether the different foraging challenges were overcome with differing reliance on social learning, we fit each model with a parameter that constrained social transmission rates to either be different or the same between prey types [57].

Each type of learning model (i.e. asocial, social additive, multiplicative and no-ILV models, fit with the assorted combinations of ILVs, where social transmission was constrained to be either the same or different between prey types) was fit with a constant baseline rate of asocial learning, and, in a different run of the models, with a baseline asocial learning rate that, in turn, had been fit with a function correspondent to a gamma distribution [71,75]. Models with the gamma baseline function were included to control for potential changes in asocial learning rates over the course of the prey extraction trials [71,75]. All models with each type of baseline function were then fit to the association networks described above, where associations between the otters were heterogeneous—henceforth referred to as ‘social networks’ [71,75–77]. All models with each type of baseline function were also fit to ‘group networks’, where all association indices between group members were fixed to equal 1, and were therefore homogeneous [76–79]. This was to ascertain whether newly learned prey interaction and meat extraction behaviours diffused by means of the heterogeneous associations in the otter groups' social networks, or uniformly through the group [76–79].

We summed the Akaike weights for each individual model to quantify the relative support for each model type, with each baseline asocial learning rate (i.e. constant or gamma) fit to each network (i.e. social or group). We then used the summed Akaike weights to compare asocial learning models to social learning models (i.e. social additive, multiplicative, and no-ILV models), as well as social learning models with the same versus different rates of social transmission for the three natural prey types. All individual models were ranked based on AICc, and the top set was made up of models within ΔAICc less than or equal to 2 of the overall best-supported model [80]. Here, we report the social transmission rate estimates per unit of network connection relative to the baseline rate of asocial learning (henceforth referred to as ‘social transmission rate’) from all the models in the respective top sets.

#### 2.6.2. Are Asian short-clawed otters faster at extracting familiar food from artificial puzzles or meat from unfamiliar natural prey items?

We tested whether otters took more time to extract meat from natural prey types than they did to extract food rewards from artificial foraging tasks using a GLMM with a gamma error structure and log link function (R package ‘lme4’; [81]), due to positively skewed data. The response variable was ‘extraction time’: the amount of time (in seconds) that otters spent interacting with tasks and prey items before extracting food from them for the first time. The explanatory variables were challenge type (‘fish’, ‘crabs’ and ‘mussels’, and tasks 1 to 5, coded as factors), the group to which otters belonged (i.e. Newquay or Tamar), sex (females coded as ‘1’ and males coded as ‘0’) and otter age (adults, i.e. otters aged greater than or equal to 1 year, coded as ‘1’, and pups, i.e. otters aged less than 1 year, coded as ‘0’). To control for repeated measures of the same individuals, each otter's identity was included as a random effect. Each combination of explanatory variables was evaluated and ranked by Akaike's information criterion, corrected for small sample size (AICc; [80]), via subset selection of the ‘maximal model’ that contained all the explanatory variables (R package ‘MuMIn’; [82]). Then, once more complex iterations of nested models had been removed in order to retain only simpler models with stronger statistical support, the ‘top set’ was formed of models within ΔAICc ≤ 2 of the best-supported model (i.e. the model with the lowest AICc value, following the ‘nesting rule’; [83]). As our top set consisted of just one model, we report only the results from this best-supported model here [80].

## 3. Results

### 3.1. Do Asian short-clawed otters copy each other when first interacting with natural prey items?

All of the otters, except the oldest individual in the Newquay group (aged 9 years), interacted with all three natural prey types (electronic supplementary material, table S3). The best-supported TADA models analysing the time sequence in which otters interacted with natural prey were multiplicative models with gamma asocial learning rate baselines, fit to the homogeneous ‘group’ networks, where the rate of social transmission was different between prey types (Akaike weight: 33.24%; table 1). All but one of the top set models were multiplicative (electronic supplementary material, table S4). The strong support for the multiplicative models suggests that the otters' first interactions with the three prey types were due to a combination of social and asocial learning. All top set models were also all fit to the ‘group’ networks (electronic supplementary material, table S4). This indicates that, where otters did use social learning, they did not preferentially copy individuals they were closely associated with.

**Table 1.** A comparison of the relative support (i.e. percentage of overall support based on summed Akaike weights) for different social and asocial learning models with gamma baselines, fit to group (i.e. homogeneous associations between individuals) and social (observed heterogenous associations between individuals) networks, for the first instance each otter interacted with, and extracted food from, the natural prey types. *Italicized* values indicate the model types with the most statistical support for each information type. Electronic supplementary material, table S6 reports the relative support for models fit with constant baselines.

model type	rate of social transmission between tasks	percentage of overall support based on summed Akaike weights	
		interaction	extraction
asocial		<0.01	<0.01
group network			
additive	same	9.21	1.73
	different	10.87	22.93
multiplicative	same	19.95	1.40
	different	*33.24*	20.90
no ILVs	same	7.91	1.70
	different	9.08	*28.58*
social network			
additive	same	0.88	0.25
	different	1.54	1.91
multiplicative	same	1.59	0.21
	different	3.68	1.77
no ILVs	same	0.76	0.25
	different	1.28	2.40

**Figure 1.**
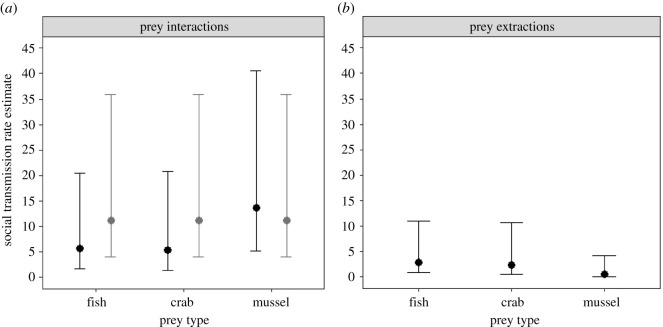
The social transmission rates per unit network connection relative to the baseline rate of asocial learning, as estimated by (*a*) the top model of first prey interactions where social transmission rates were constrained to be different between the three natural prey types (black) and the top model where social transmission rates were constrained to be the same across the three natural prey types (grey), and (*b*) the top model analysing the time sequence in which otters extracted the meat from the prey types. Error bars indicate 95% confidence intervals. Social transmission rate estimates between models in the respective top sets were relatively similar (tables 2 and 3), and we have plotted these model estimates for illustration.

Three of the four top set models also indicated that the extent to which otters relied on social learning was different between the prey types (electronic supplementary material, table S4), in that the otters' social information use was similar when they were first interacting with the fish and crabs, but higher (relative to the other prey items) when they were interacting with the mussels (figure 1*a* and table 2). That being said, the 95% confidence intervals for the prey-specific social learning rate estimates overlapped in each of these models (figure 1*a* and table 2). Furthermore, the remaining top set model denoted that social transmission was the same between prey types. Therefore, although the otters may have had a tendency to rely more on social information when learning to extract meat from the mussels, we cannot be certain whether this is indeed the case. This uncertainty is also reflected in the percentages of the first interactions with prey types that occurred due to social learning (excluding the ‘innovator’), as estimated by the three ‘different social learning models' in the top set, which were all virtually identical across the three prey types (table 2). Finally, although top set models fitted with age and sex indicated that adult and male otters interacted with the prey types sooner than pups and females, respectively, we cannot be certain that these variables had a robust effect on otters' learning to interact with the natural prey types, as a model not fit with either ILV was in the top set as well (table 2).

**Table 2.** Social transmission rate estimates for when otters were first learning to interact with the natural prey types, the percentage of first interactions with natural prey types that occurred due to social learning (excluding the first otter to interact with the prey, as per definition it could not have done so through social learning) and the effects of ILVs on the rate of learning, as estimated by the top set of NBDA models. All top set models were fit to the group networks and with gamma baseline functions.

model type	model ΔAICc	estimated social transmission rate for each prey type (95% CI)			estimated percentage of first interactions that occurred due to social learning for each prey type (95% CI)			estimated effect of ILVs on learning rate (95% CI)	
		fish	crab	mussel	fish	crab	mussel	age	sex
multiplicative	0	5.71 (1.66, 20.48)	5.36 (1.42, 20.76)	13.67 (5.27, 40.63)	94.85% (85.16%, 98.47%)	94.70% (83.60%, 98.54%)	97.81% (94.62%, 99.24%)	NA	x 1.72 (1.03, 2.88)
multiplicative	1.14	11.11^a^ (4.05, 35.96)	11.11^a^ (4.05, 35.96)	11.11^a^ (4.05, 35.96)	97.30% (93.11%, 99.14%)	97.30% (93.11%, 99.14%)	97.30% (93.11%, 99.14%)	NA	x 1.60 (0.96, 2.67)
no ILV	1.76	5.89 (1.68, 21.08)	5.59 (1.45, 21.58)	13.05 (5.03, 38.46)	94.99% (85.26%, 98.51%)	94.90% (83.91%, 98.59%)	97.71% (94.40%, 99.20%)	NA	NA
multiplicative	1.92	5.22 (1.47, 19.11)	5.18 (1.37, 20.12)	13.06 (4.99, 39.07)	94.41% (83.62%, 98.36%)	94.53% (83.21%, 98.49%)	97.71% (94.34%, 99.21%)	x 1.27 (0.72, 2.30)	x 1.77 (1.05, 2.98)

^a^Indicates that the social transmission rate parameter estimate is constrained to be the same across all prey types, as denoted by the particular model.

### 3.2. Do Asian short-clawed otters copy each other when extracting meat from natural prey items?

All the otters, barring the single 1-year old adult in the Newquay group, managed to extract the meat from two of the natural prey types, with 11 of the 20 otters across the two groups managing to extract the meat from all three prey types (electronic supplementary material, table S3). The models that best explained the time sequence in which otters learned to extract meat from the natural prey types were no-ILV models, with gamma baselines fit to the group networks, where the rate of social transmission was different between task types (Akaike weight: 28.58%; table 1). The no-ILV models suggest that neither social nor asocial learning rates were affected by otter sex or age. However here, the other model in the top set was an additive model that specified adult otters were faster at extracting the meat from each prey type than otter pups (2.21× (95% CI = [0.44, 10.87]); per year of age; table 3). So although we can rule out a sex effect, whether otters' age affected the rate at which they learned to extract meat from the natural prey types is less clear. Whether otters only used social information or a combination of strategies when learning to extract and consume prey meat is also uncertain.

**Table 3.** Social transmission rate estimates for when otters were first learning to extract meat from the natural prey types, the percentage of first extractions of meat from the natural prey types that occurred due to social learning (excluding the innovator) and the effects of ILVs on the rate of learning, as estimated by the top set of NBDA models. Both models in the top set were fit to the group networks and with gamma baseline functions.

model type	model ΔAICc	estimated social transmission rate for each prey type (95% CI)			estimated percentage of first extractions that occurred due to social learning for each prey type (95% CI)			estimated effect of ILVs on learning rate (95% CI)	
		fish	crab	mussel	fish	crab	mussel	age	sex
no ILVs	0	2.94 (0.88, 11.09)	2.37 (0.56, 10.63)	0.49 (0.00, 4.19)	90.45% (75.66%, 97.15%)	88.57% (67.58%, 97.04%)	60.12% (0.00%, 91.64%)	NA	NA
additive	1.83	5.84 (0.93, 116.23)	4.82 (0.62, ∞)^a^	1.12 (0.00, 8.32)	92.17% (75.78%, 92.55%)	90.12% (68.48%, 99.99%)^a^	65.38% (0.00%, 68.62%)	x 2.21 (0.44, 10.87)	NA

^a^TADA was unable to precisely estimate the upper 95% confidence interval of the social transmission rate estimate, in the second top set model, for when otters were learning to extract the crab meat. Consequently the 95% confidence interval could also not be calculated for the estimated percentage of first crab meat extractions that occurred due to social learning (excluding the innovator).

Both top set models were fit to the group networks (electronic supplementary material, table S5), which indicates that otters learned to extract the meat from the prey items by copying their group mates equally, rather than by preferentially copying individuals they were closely associated with. The top set models also suggested that the extent to which otters relied on social learning was different between the prey types (figure 1*b* and table 3). Social transmission rate estimates indicated that the otters' use of social information and the percentage of first meat extractions that occurred due to social learning (excluding the innovator) were similar when they were first extracting meat from the fish and crabs, but slightly lower when extracting meat from the mussels (table 3). However, the 95% confidence intervals for both these social learning measures were very wide and included zero in the case of the mussels (figure 1*b* and table 3). Thus, although the results show that otters did use social information to some extent when learning to exploit the fish and crabs, whether the same was true for mussels remains unclear.

### 3.3. Are Asian short-clawed otters faster at extracting familiar food from artificial puzzles or meat from unfamiliar natural prey items?

**Figure 2.**
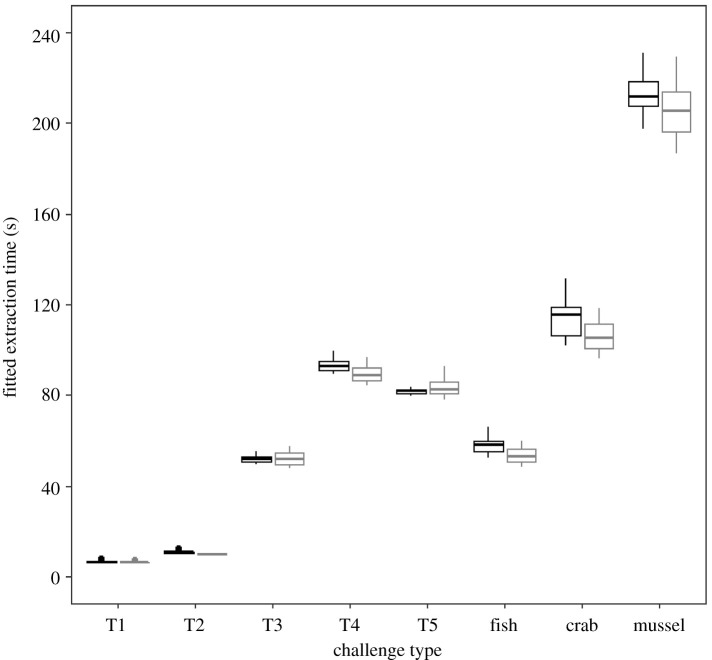
The fitted extraction times from the best-supported extraction time model showing how the amount of time (in seconds) that otters spent extracting meat from the artificial foraging tasks and natural prey types was affected by challenge type (‘T’ denotes ‘task’ on the *x*-axis labels). Black boxes denote fitted extraction times from otters in the Newquay group, and the grey boxes represent fitted extraction times from otters in the Tamar group. The bold line within each box indicates the 50th percentile and the top and bottom of each box signify the 75th and 25th percentiles, respectively. The whiskers signify the highest and lowest values that are not outliers. Outliers are represented by the points above the boxes. For a plot of the raw extraction times, please see electronic supplementary material, figure S2.

The time it took otters to extract food from the foraging challenges was only affected by the specific challenge type they were attempting to exploit (table 4): it took otters longer on average to extract the meat from natural and artificial prey types with more complex protective measures (figure 2). However, the fish, which was the only challenge type without any physical protective measures, did not take otters the least amount of time to exploit (figure 2). Instead, the average extraction times suggest that fish was actually the fourth most difficult foraging challenge to overcome after artificial tasks 1, 2 and 3, although extraction times for task 3 and fish were very similar (mean ± s.d. of the fitted extraction times, in seconds, from the top set model; task 1: 6.44 ± 0.60 s; task 2: 10.25 ± 0.99 s; task 3: 51.95 ± 2.92 s; fish: 55.09 ± 4.64 s). Tasks 5 and 4 took the otters the next longest to overcome, respectively (task 5: 82.73 ± 4.49 s; task 4: 91.39 ± 4.52 s). Finally, the otters spent the most time extracting the meat from the crabs and mussels (crabs: 109.02 ± 9.41 s; mussels: 206.50 ± 13.65 s; figure 2).

**Table 4.** Model selection table for variables affecting the time (in seconds) otters spent extracting familiar rewards from novel artificial tasks and meat from unfamiliar natural prey types. Explanatory variables in the models were the challenge type (i.e. tasks 1 to 5 and the three prey types), the group to which otters belonged (i.e. Newquay or Tamar), otter age (adults, i.e. otters aged greater than or equal to 1 year, coded as ‘1’, and pups, i.e. otters aged less than 1 year, coded as ‘0’) and sex (females coded as ‘1’ and males coded as ‘0’). The best-supported model retained in the top set (in *italics*) after application of the ‘nesting rule’ and the null model is reported.

fixed effects	intercept	d.f.	logLik	AICc	ΔAICc	adj. weight
∼ *challenge type*	*4*.*69*	*10*	*−502*.*40*	*1027*.*23*	*0*.*00*	*1*.*00*
∼ 1	4.42	3	−546.56	1099.35	72.12	0.00

## 4. Discussion

### 4.1. Do Asian short-clawed otters copy each other when learning to exploit natural prey items?

When otters first encountered the natural prey types, our results suggest they used a combination of social and asocial learning when deciding to interact with them. Whereas, when learning to extract and consume prey meat, our NBDA could not decisively determine whether they used social information only or a combination of asocial and social learning. Nevertheless, social transmission rate estimates were lower when otters were first extracting meat from hard-shelled prey types (i.e. the crabs and mussels), than when they were first interacting with them. The result here mirrors our previous findings, where these same otters relied more on social learning when interacting with artificial foraging tasks than when they were extracting familiar food rewards from them [57]. Moreover, social transmission rates estimated here for the extraction of natural prey items were markedly similar to those we reported for otters learning to extract meatballs from the artificial tasks [57]. Although Asian short-clawed otters do not appear to forage cooperatively in the wild, they are known to forage along riverbanks and in wetlands within view of their group mates [38,52,54]. Results here support our previous interpretation that Asian short-clawed otters may be drawn to food patches, and learn what types of prey to exploit, by observing their conspecifics foraging around them. However, once prey items have been discovered, otters may learn how to overcome any protective measures mainly asocially [57], regardless of whether they are familiar with the food reward within.

Despite the fact that our models could not decisively establish whether the otters' reliance on social learning was the same or different across natural prey types, our results seem to indicate that otters relied on social information more when they were learning to interact with the natural prey types compared to the artificial foraging tasks. The social transmission rate estimates for when otters were learning to interact with the prey types were approximately twice as high in the ‘same social learning model’ in the top set here as compared to the estimates for the artificial foraging tasks from our previous study [57]. This may be because otters were familiar with the palatability of the meatballs and the concept of extracting food from artificial food puzzles, having been presented with similar tasks as part of captive enrichment programmes. Furthermore, the otters had both visual and olfactory access to the familiar meatballs through the transparent, perforated puzzle boxes, while the meat inside the hard shells of the crabs and molluscs was both less visible and unfamiliar. The otters thus had to learn about the palatability of the natural prey items, as well as how to extract to meat from them, which may explain why they more readily looked to their group mates for information.

The fact that the otters were unfamiliar with the natural prey types may also have made them more motivated to gather information quickly as compared to when they tackled the artificial tasks and meatballs. This may help explain why they appeared to learn indiscriminately from their group mates rather than showing a preference for particular group members. These results contrast with our previous work, where otters preferentially copied their close associates when learning to interact with, and extract the rewards from, the artificial foraging tasks [57].

### 4.2 Are Asian short-clawed otters faster at extracting familiar food from artificial puzzles or meat from unfamiliar natural prey items?

Otters were faster at extracting the food rewards from the puzzles than the meat from the natural prey types that presented an extractive challenge, i.e. the crabs and mussels. Otters in both groups spent time manipulating these natural prey items with their paws, carrying them around the enclosure, and at times appearing to ‘juggle’ them, similarly to how they are known to do with stones [38–40,42], before attempting to extract the meat from them. By contrast, when presented with the artificial foraging tasks, otters began to try and access the meatballs, which they were regularly fed, straight away. We suggest this is because otters were able to skip the exploratory phase of determining the palatability of the meatballs, given that they already recognized them as food. Whereas being unfamiliar with the crabs and mussels meant otters had to first spend time determining that these prey items contained palatable meat before attempting to extract it.

Further to the otters' familiarity with the palatability of the meat within each type of extractive foraging challenge, their degree of familiarity with the protective measures themselves could have been another potential factor contributing to the faster task extraction times. Both otter groups had previously been exposed to artificial food puzzles constructed from Tupperware boxes as part of captive enrichment programmes (although of a different design to those we presented here; see §2.4.1 for details). Given that these otters can remember the solutions to extractive food puzzles for months after first being exposed to them [57], they could have remembered relevant skills learned during these past presentations and applied them here. Thus enabling them to extract task rewards faster than the meat from the hard-shelled prey types, the protective measures of which they were naïve to. This in turn is indicative that further to learning that certain physically protected prey items are palatable, otters must also learn specific techniques to overcome the protective measures of such prey items. This is also the case in other small mammal species that feed on physically protected prey items such as meerkats (*Suricata suricatta*). Where juveniles learn from adults to use their front paws and teeth to dismember scorpions (genus Parabuthus) in order to avoid potentially lethal stings during consumption [84].

Our suggestion above that otters’ familiarity with food items affected how long they spent interacting with them before starting to eat them, initially appears to be refuted by the lack of a meaningful difference in fish ‘extraction’ times between the two otter groups (figure 2). This is due to the fish being the only food item, across both the tasks and prey types, that differed in novelty between our study groups, with the Tamar otters having been fed fish on a non-regular basis. If food item familiarity did indeed dictate ‘extraction’ time, we might expect otters in the Tamar group to begin eating the fish sooner after first interacting with them compared to the Newquay otters. However, as the fish was the only challenge type for which otters did not have to overcome an unpalatable barrier to access the meat, otters in the Newquay group were able to quickly ascertain that the unprotected fish meat was edible. Thereby negating the advantage possessed by the Tamar group from having had previous experience with this prey type. In fact, unlike with the crabs and mussels (see above), otters in both groups quickly began trying to bite off pieces of fish upon first interacting with them. With this being the case, it is somewhat surprising that otters did not take the least amount of time to actually start consuming the fish meat. Although Asian short-clawed otters are known to feed on small fish in the wild, they make up a relatively small percentage of their overall diet, compared to crustaceans and molluscs. Consequently, their dentition is specialized for breaking open the hard shells of these prey types, rather than for slicing through the soft flesh of fish [38,52,54]. The otters here could therefore have struggled to quickly bite off pieces of fish small enough to consume, which precipitated the surprisingly long ‘extraction’ times.

### 4.3. Do age and sex affect how Asian short-clawed otters learn about exploiting natural prey items?

Our results are inconclusive regarding the effects of age and sex on the rate at which otters learned to interact with, and extract the meat from, the natural prey types; models containing a variety of ILV combinations, including models with neither ILV, were present in both NBDA top sets (tables 2 and 3). That being the case, we can draw some cautious inference from the prevalence of each ILV in the respective top sets. Three of the four top models indicated that male otters learned to interact with novel prey types faster than females (table 2). It is thought that male Asian short-clawed otters are mainly solitary in the wild and only temporarily join social groups consisting of a dominant female and her offspring during the breeding season [38]. It could be the case, therefore, that male otters are inherently faster learners, particularly in the context of interacting with new food sources, as in the wild they often cannot rely on group mates to direct them towards new profitable foraging patches or food sources.

Both NBDA top sets also contained models which denoted that adults learned to interact with, and extract meat from, novel prey types faster than pups. Moreover, in all but two cases the first otters to interact with and extract meat from the prey types (i.e. the innovators of these behaviours) were adults. We therefore suggest that adult otters first learned to interact with, and extract the meat, from novel prey types, and subsequently acted as demonstrators to the pups who then copied these behaviours (although to a lesser extent when extracting the meat from the prey types than when interacting with them; see §4.1). Adults acting as the demonstrators of foraging behaviours for younger individuals has been observed in other otter species; both Eurasian and sea otters pups are thought to learn where and what to hunt, as well as prey capture techniques, by observing the behaviours of their mothers [43,49–51]. In Eurasian otters, there is also evidence to suggest that mother otters facilitate the learning of prey capture techniques by dropping live prey items, such as fish, into rock pools for their young to recapture [50,51]. Our results suggest that Asian short-clawed otters may be another species where adults act demonstrators of novel foraging behaviours.

### 4.4. Wider implications

Our findings and those of our previous study [57] suggest that captive Asian short-clawed otters learn to interact with both artificial and natural unfamiliar food sources by observing their group mates, while they learn to how to extract meat from them mainly asocially. However, our studies were conducted on the same two groups of captive otters. It therefore remains to be established whether patterns of social learning observed here are prevalent species wide, across captive otters only, or if they are specific to the two captive groups studied here. To determine the generalizability of these findings, ideally these experiments would be replicated on more captive groups and where possible, on wild otters, across a variety of otter species. Our finding that otters' familiarity with the palatability of the meat within extractive foraging challenges affected how long it took them to overcome the physical protective measures should also be interpreted with caution, given that the artificial and natural foraging challenges differed in several aspects. For example, the otters had prior experience with Tupperware puzzle boxes as part of enrichment programmes, but not with natural prey types similar to crabs and mussels (see §4.2). Furthermore, otters were able to see the meatballs through the clear boxes, while the meat within the physically protected natural prey types was not readily visible. To draw more robust conclusions regarding the influence of food familiarity on extractive foraging performance, one could present otters with one set of clear puzzles that contain a known food reward and another set that house an unknown food reward. Otters would be able to see both reward types and have to overcome the same kind of protection to access them.

While presenting two different types of foraging challenges means we must show caution when interpreting differences in extractive foraging performance between them, presenting natural prey to captive otters has given us an indication of how they fare when they encounter such prey items for the first time, and how they learn to exploit them. This in turn allows us to identify how captive Asian short-clawed otters may cope with one of the key challenges of living in the wild, which could prove crucial if reintroductions are adopted as a method for their conservation in the future. Such reintroductions often involve the release of animals born and raised in captivity into the wild to help re-establish species of conservation concern, and aid in the recovery of their wild populations [58,59]. However, if captive otters, unfamiliar with their natural prey, initially struggle to recognize them as food, it could hinder reintroduction success. Assessments of species reintroductions have shown that behavioural deficiencies among individuals in reintroduced sub-populations commonly result in high mortality rates [58,85–88]. For example, the success of golden lion tamarin (*Leontopithecus rosalia*) reintroductions was hampered because tamarins, among other behavioural deficiencies, were unable to recognize their natural food items and so starved to death [86].

Though captive Asian short-clawed otters initially struggled to recognize and overcome the extractive challenges presented by natural prey items, we also demonstrated that they readily learn how to do so via asocial and social learning. Knowledge of their use of these learning strategies could be exploited to design reintroduction protocols and prepare captive individuals for release to the wild. Their employment of social information when learning to interact with novel prey items may prove particularly useful. For example, Asian short-clawed otters could be reintroduced in social groups formed around an informed adult demonstrator otter, from whom naïve otters may more quickly learn what to forage for in the wild. Such pre-release training has been shown to be an effective tool to maximize reintroduction success [58,59]. Research into another mustelid species, the black-footed ferret (*Mustela nigripes*), suggests that captive individuals presented with live prey items, including their natural prey—white tailed prairie dogs (*Cynomys leucurus*)—displayed higher predatory proficiency when released into the wild [87,89–91].

At present the IUCN Red List of Threatened Species reports that habitat protection has somewhat slowed Asian short-clawed otter population declines, but that sustained conservation efforts are nevertheless essential for the long-term survival of this ‘Vulnerable’ species [52,56]. We therefore suggest that Asian short-clawed otter reintroductions into protected areas with abundant natural prey may be a viable conservation strategy, as long as the reintroduced individuals have been appropriately prepared to overcome the challenges they will face in the wild. However, further investigations into the practicality of such measures, and how they would work in tandem with existing *in situ* conservation actions, would be needed to confirm the feasibility of such a strategy.

To conclude, our findings indicate that captive Asian short-clawed otters took longer to extract the meat from unfamiliar natural prey types than familiar rewards from mechanistically novel artificial food puzzles. However, their capacity for learning, both socially and asocially, to tackle natural extractive foraging challenges suggests that if they are appropriately prepared, reintroductions of captive-raised otters into the wild could be a viable conservation action plan.

## Data Availability

The data and R code used in the GLMM and NBDA analyses have been uploaded as part of the electronic supplementary material [93]. Model selection tables for the NBDA, and tables detailing otter group compositions, descriptions of the novel foraging tasks, and otter prey interactions and extractions, in addition to figures depicting otter group association networks, have been uploaded as part of the electronic supplementary material.
